# Comparative advantages of Zn–Cu–In–S alloy QDs in the construction of quantum dot-sensitized solar cells†

**DOI:** 10.1039/c7ra12321c

**Published:** 2018-01-18

**Authors:** Liang Yue, Huashang Rao, Jun Du, Zhenxiao Pan, Juan Yu, Xinhua Zhong

**Affiliations:** School of Chemistry and Molecular Engineering, East China University of Science and Technology Shanghai 200237 China zhongxh@ecust.edu.cn +86 20 8528 0319 +86 20 8528 0319; College of Materials and Energy, South China Agricultural University 483 Wushan Road Guangzhou 510642 China zxpan@scau.edu.cn

## Abstract

Alloyed structures of quantum dot light-harvesting materials favor the suppression of unwanted charge recombination as well as acceleration of the charge extraction and therefore the improvement of photovoltaic performance of the resulting solar cell devices. Herein, the advantages of Zn–Cu–In–S (ZCIS) alloy QD serving as light-harvesting sensitizer materials in the construction of quantum dot-sensitized solar cells (QDSCs) were compared with core/shell structured CIS/ZnS, as well as pristine CIS QDs. The built QDSCs with alloyed Zn–Cu–In–S QDs as photosensitizer achieved an average power conversion efficiency (PCE) of 8.47% (*V*_oc_ = 0.613 V, *J*_sc_ = 22.62 mA cm^−2^, FF = 0.610) under AM 1.5G one sun irradiation, which was enhanced by 21%, and 82% in comparison to those of CIS/ZnS, and CIS based solar cells, respectively. In comparison to cell device assembled by the plain CIS and core/shell structured CIS/ZnS, the enhanced photovoltaic performance in ZCIS QDSCs is mainly ascribed to the faster photon generated electron injection rate from QD into TiO_2_ substrate, and the effective restraint of charge recombination, as confirmed by incident photon-to-current conversion efficiency (IPCE), open-circuit voltage decay (OCVD), as well as electrochemical impedance spectroscopy (EIS) measurements.

## Abstract 2

Benefiting from the accelerative electron injection and retarded charge recombination, Zn–Cu–In–S alloy QD based QDSC achieved a PCE of 8.55%, which is 21%, and 82% higher than those of CIS/ZnS, and pristine CIS QDs based solar cells, respectively.

## Graphical abstract

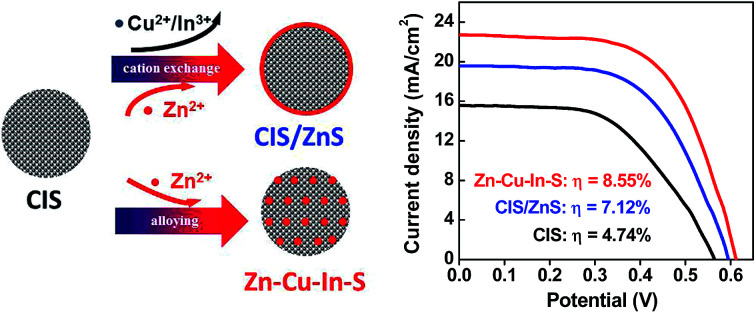

## Introduction

Colloidal semiconductor quantum dots (QDs) are appealing light-harvesting materials for optoelectronic conversion applications due to their solution processability as well as their distinguished optoelectronic properties.^1–3^ The quantum dot-sensitized solar cell (QDSC), in which QDs are deposited on a metal oxide (typically TiO_2_) film, is believed to be a promising candidate for the next-generation solar cells.^3^ Among various QDs, I–III–VI_2_ group (especially CuInS_2_ (CIS), and CuInSe_2_ (CISe)) QDs have drawn special attention as sensitizers due to their environmental benignity, high absorption coefficient (∼10^5^ cm^−1^), and near optimal band gap (1.0–1.5 eV) for photovoltaic applications.^4–10^ Very recently, a new efficiency record over 11% for QDSCs has been reported based on a CISe QDs sensitizer.^11^ In comparison, the photovoltaic performance of its counterpart CIS-based QDSCs is much poorer with the highest certified efficiency of only 6.66%.^12^

The relatively low performance of CIS based QDSCs is at least partially ascribed to the presence of trap state defects in CIS QD itself, which results in both charge recombination inside QDs (before electron injection into the metal oxide substrate) and at interfaces between metal oxide substrate and QD (after electron injection).^13–16^ To eliminate/minimize trap-state defects in QD sensitizers and suppress the related charge recombination, overcoating an inorganic shell with wider band gap (in particular ZnS) around QDs to form type-I core/shell structure is a well-established approach.^12,17–21^ Unfortunately, in spite of the beneficial suppression of charge recombination dynamics, the formed shell layer with wide band gap in this core/shell structure would retard the charge extraction rate simultaneously, and deteriorate the photovoltaic performance accordingly.^12,22^ Furthermore, the preparation procedure for a core/shell structured QD is usually time-intensive. As for other surface passivation method, it is common to implement photoanode post-treatment by depositing ZnS layer over the sensitized electrode *via* successive ionic layer adsorption and reaction (SILAR) route to reduce the TiO_2_/QD/electrolyte interfaces charge recombination and consequently improve the photovoltaic performance of the cell devices.^23–27^ However, the retardation of charge recombination based on SILAR route is less efficient because ZnS layer is deposited as a loose particle-packing network around the exposed surface of QD sensitizers and therefore cannot effectively minimize the intrinsic defects inside QDs. Meanwhile, the post-deposited ZnS layer by SILAR route cannot serve as an energetic barrier layer at TiO_2_/QD interface to reduce the charge recombination at these interfaces.^16,28–32^

In contrast, the alloyed structure could outperform the type-I core/shell configuration, because an alloying process can lead to (at least partially) homogeneous electronic structure rather than create discrete higher energy levels around QD surface.^22,33–36^ Targeting on optimizing the electronic structure in CISe QDs to match TiO_2_ substrate and accelerate the photogenerated electrons injection rate, impressive results have been achieved based on the alloying strategy.^11,29,37–39^ Among these, with the incorporation of ZnSe into CISe QDs, the density of trap state defects in the resulting Zn–Cu–In–Se (ZCISe) was reduced, and the corresponding charge recombination dynamics was suppressed.^11^ As a result, the ZCISe based QDSC has achieved a record PCE of 11.6% with use of titanium mesh anchored mesoporous carbon (MC/Ti) counter electrodes.^11^ Meanwhile, the simultaneous incorporation of foreign additives as alloying component during QD synthesis can undoubtedly simplify experimental process. These expectations prompt us to further promote the photovoltaic performance of CIS-based QDSCs by employing alloying strategy.

In this work, the electronic structure of CIS QDs was tailored by incorporation of ZnS component into CIS host QDs to form Zn–Cu–In–S (ZCIS) alloy QDs. The comparative advantages of ZCIS alloy QD serving as light absorber in the construction QDSC were studied relative to previously adopted core/shell configurated CIS/ZnS, and pristine CIS QDs under their optimal conditions. Benefiting from the higher electron injection rate and efficient inhibition of charge recombination dynamics, the ZCIS alloy based QDSCs exhibit the best PCE of 8.55%, which exceeds 21%, and 82% over those derived from CIS/ZnS and CIS QDSCs, respectively.

## Experimental section

### Chemicals

Zinc acetate (Zn(OAc)_2_, 99.99%), indium acetate (In(OAc)_3_, 99.99%), oleylamine (OAm, 95%), 1-octadecene (ODE, 97%), sulfur powder (99.99%) were purchased from Aldrich, copper iodide (CuI, 99.99%), 3-mercaptopropionic acid (MPA, 99%) were obtained from Alfa Aesar. All reagents were used as received without further treatments.

### Synthesis of oil-soluble QDs

The oil-soluble 4.3 nm CIS QDs and CIS/ZnS core/shell QDs with 4.3 nm CIS core and 0.7 monolayer of ZnS shell were synthesized according to literature method.^12^ For CIS QDs preparation, 0.1 mmol CuI and 0.1 mmol In(OAc)_3_ were loaded in a 50 mL three-neck flask containing 2.0 mL OAm and 4.0 mL ODE. The resulting mixture solution was subsequently heated to 90 °C and keep vacuum condition for 10 min to remove air and low-boiling impurities. After the solution was heated up to 160 °C under nitrogen atmosphere, 0.4 mL 1.0 M OAm-S precursor solution (0.16 g sulfur was dissolved in 5.0 mL OAm beforehand) was injected into the reaction system and stayed for 30 min. The obtained CIS QDs were purified by precipitation and centrifugation procedure with use of ethanol and acetone. The purified CIS QDs were dispersed in 2.0 mL OAm and 4.0 mL ODE and degassed under vacuum at 40 °C for 20 min. For synthesis of CIS-Z QDs, the above solution was then heated to 100 °C and 0.4 mL 0.1 M Zn(OAc)_2_ stock solution (obtained by dissolving 0.44 g Zn(OAc)_2_ in 1.6 mL OAm and 18.4 mL ODE) was injected into mixture solution and kept at set temperature for 30 min to ensure enough cation exchange. It is noted that all of the stock solution were stored under ambient condition.

The preparation of OAm-capped Zn–Cu–In–S (ZCIS) QDs was similar to that of pristine CIS QDs. Briefly, 0.1 M of Zn(OAc)_2_ precursor solution was prepared by dispersing Zn(OAc)_2_ in a mixture of OAm and ODE with a volume fraction of 1 : 4. A certain amount (0–0.6 mL, 0.1 M) of Zn(OAc)_2_ precursor solution was added into the mixture solvent containing CuI (0.1 mmol), In(OAc)_3_ (0.1 mmol), OAm (2 mL), and ODE (1.5 mL) in a 50 mL three-necked flask. The mixture solution was degassed at 90 °C for 10 min and then heated up to 160 °C under N_2_ atmosphere. The oil-soluble Zn–Cu–In–S QDs were obtained by rapidly injecting 0.4 mL of 1.0 M sulfur precursor solution (prepared by dissolving sulfur powder in OAm) into the reaction system at 260 °C, followed by staying at this temperature for another 5 min.

### Preparation of water-soluble QDs by ligand exchange

The above prepared oil-soluble QDs were transferred into aqueous solution *via* a ligand exchange procedure with use of MPA as a phase conversion reagent as described in our previous work.^40^ Generally, the oil-soluble QDs were purified by precipitation and centrifugation procedure with addition of excessive ethanol and acetone. Then, the QDs were dispersed in 20.0 mL CH_2_Cl_2_ and a MPA-methanol solution (2.0 mmol MPA in 1.0 mL methanol media with pH = 11) was added into the QD-CH_2_Cl_2_ solution under stirring for 3 min. Afterwards, 20 mL of deionized water was added into the above QD mixture and stirred for another 2 min. Finally, QDs were transferred into the superincumbent water phase from the underlying CH_2_Cl_2_. After precipitation with use of acetone, the resulting QDs were re-dispersed in 1.0 mL of deionized water and adjusted pH to 10 with 30% NaOH.

### Assembly of solar cell devices

TiO_2_ mesoporous film electrodes (20 ± 0.5 μm transparent layer and 5 ± 0.5 μm light scattering layer on FTO) were prepared by screen printing method according to our previous report.^41^ For the deposition of QDs, 40 μL of the above prepared MAP-capped QDs aqueous solution was pipetted on the TiO_2_ film and stayed for 2 h at 50 °C before rinsing sequentially with water and ethanol. Then, the QD sensitized photoanodes were overcoated with ZnS layers by alternately immersing them into 0.1 M Zn(OAc)_2_ methanol solution, and 0.1 M Na_2_S aqueous solution for 6 cycles with 1 min per dip, and rinsed with deionized water and ethanol between each dip.^42,43^

The solar cells were assembled with Cu_2_S/brass counter electrode (prepared by immersing the brass foil into 1.0 M HCl at 90 °C for 1 hour), and QD-sensitized TiO_2_ photoanode with a binder clip separated by a 50 mm thick Scotch spacer. Polysulfide aqueous solution electrolyte (2.0 M Na_2_S, 2.0 M S, and 0.1 M KCl) was injected between the two electrodes with a hole pre-drilled on the counter electrode.

### Characterization

The UV-vis absorption and photoluminescence (PL) emission spectra were respectively carried out on the UV-visible spectrophotometer (Shimadzu UV-3101 PC) and a fluorescence spectrophotometer (Cary Eclipse Varian). Incident photon-to-current conversion efficiency (IPCE) spectra was obtained on the Keithley 2000 multimeter under a special product DK240 monochromator with a 300 W tungsten lamp. The cross section image of TiO_2_ film was observed with scanning electron microscope (SEM, FEI NOVA Nano SEM450). Transmission electron microscopy (TEM) images were obtained by using a JEOL JEM-2100 microscope at an accelerating voltage of 200 KV. X-ray diffraction (XRD) pattern was acquired from a Siemens D5005 X-ray powder diffractometer. The X-ray photoelectron spectroscopy (XPS) spectra were performed on an ESCALAB 250Xi spectrometer. Electrochemical impedance spectroscopy (EIS) was measured by an impedance analyzer (Zahner, Zennium) under dark condition at an applied bias from −0.3 V to −0.6 V applying a 20 mV AC sinusoidal signal over the constant applied bias with the frequency ranging from 1 MHz to 0.1 Hz. Intensity-modulated photocurrent and photovoltage spectra (IMPS/IMVS) were collected using the same electrochemical workstation (Zahner) with a frequency response analyzer under intensity-modulated (30–150 W m^−2^) blue light emitting diode (457 nm) driven by zahner (PP211) source supply. Open-circuit voltage decay (OCVD) was also performed on the same Zahner electrochemical workstation, and cells were illuminated by a white LED with intensity of 100 mW cm^−2^. Current–voltage (*J*–*V*) curves were obtained from a Keithley 2400 source meter and an AM 1.5G solar simulator (Oriel, Model no. 91160) with illumination intensity of 100 mW cm^−2^.

## Results and discussion

### Optimization of Zn/Cu–In ratio and optical properties of Zn–Cu–In–S alloyed QDs

Alloying process is an effective approach to decrease the density of trap-state defects in QD materials and suppress the trap state related charge recombination, therefore improve the photovoltaic performance, especially the open-circuit voltage (*V*_oc_) in the resultant cell devices. In the synthesis of Zn–Cu–In–S (ZCIS) alloyed QDs, oleylamine (OAm) ligand with a mediate coordinating capacity was chosen as both capping agent and reaction media. Oil-soluble Zn–Cu–In–S QDs were synthesized *via* a “hot-injection” method by injecting OAm-sulfur (sulfur powder was dissolved in oleylamine in advance) precursor solution into the oleylamine reaction media containing the cationic precursor of Zn(OAc)_2_, CuI and In(OAc)_3_ at pre-set temperature. It should be noted that the relative amount of Cu, In, and S precursors was fixed at a molar ratio of 1 : 1 : 3, and the Zn/Cu–In molar ratio varied from 0 to 0.6. The detailed synthetic procedure was described in the Experimental section. Besides the ZCIS alloy QDs with different Zn contents, the core/shell structured CIS/ZnS QDs corresponding to the best photovoltaic performance were also prepared according to literature method.^12^

**Fig. 1:**
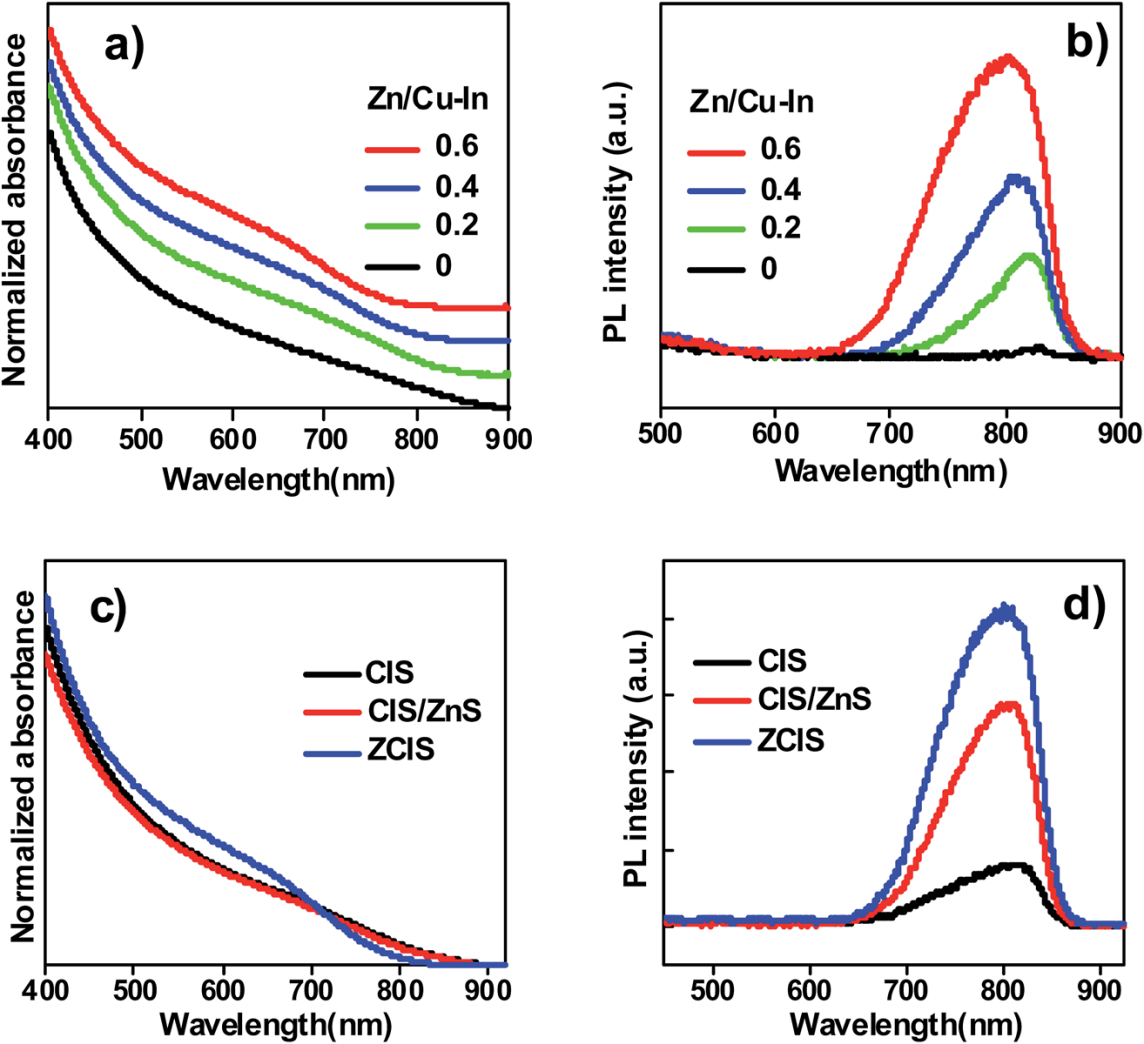
(a) Absorption spectra and (b) PL emission spectra of ZCIS QDs with various Zn/Cu–In ratios; (c) absorption spectra and (d) PL emission spectra of CIS, CIS-Z and ZCIS QDs dispersed into petroleum.

Through a facile “simultaneous nucleation and growth” approach, both the absorption and PL profiles of the obtained ZCIS alloy QDs could be expediently regulated and controlled by altering the nominal amount of Zn precursor used in the synthesis. As shown in Fig. 1a, with the fixation of the molar ratio of Cu, In, and S precursors at 1 : 1 : 3, the absorption onset of the obtained ZCIS alloyed QDs could be tuned from 870 to 830 nm by varying the Zn precursor amount from 0 to 0.06 mmol (corresponding to Zn/Cu–In molar ratio varying from 0 to 0.6). Accordingly, the PL peak position also exhibited a blue-shift from 840 to 800 nm with the increase of Zn/Cu–In molar ratio from 0 to 0.6 as shown in Fig. 1b. Furthermore, with the increase of Zn content, the photoluminescence emission intensity of the resultant alloy QDs showed a heavy dependence on the molar ratio of Zn/Cu–In (*i.e.* with the increase of Zn content, the corresponding PL intensity increased accordingly). This behavior indicates that the incorporation of Zn in CIS host favors the decrease of surface trap defects density.

For comparison, the optical spectra (including both absorption and PL emission spectra) of ZCIS alloyed QD, plain CIS, and CIS/ZnS core/shell QDs are shown in Fig. 1c and d. Similarly, as shown in Fig. 1a, a slight blue-shift existed in ZCIS QDs. This can be attributed to a part of Zn^2+^ replacement for In^3+^ and Cu^+^.^46^ Although core/shell structured CIS/ZnS and pristine CIS QDs show nearly identical light absorption range, the PL intensity of CIS/ZnS QDs was enhanced profoundly compared with that of pristine CIS QD, which is close to zero. It is well acknowledged that high density of surface trap state defects brings forward low PL emission efficiency, and lead to serve charge recombination and therefore damage photovoltaic performance of the resultant cell devices.^7,44^ The ZnS layer around CIS/ZnS QDs can reduce the defect density of CIS sensitizer and effectively restrain unwanted electron loss or charge recombination among TiO_2_/QD/electrolyte interfaces, as proven by CIS/ZnS PL spectra as shown in Fig. 1d.^12,24,25^ However, the incorporation of ZnS passivation layer around pristine CIS QD only repaired external trap-state defects rather than internal defects inhered in sensitizer material. Alloying Zn element into CIS QD is an effective method to solve this problem.^45–48^ As observed in Fig. 1d, the PL efficiency of the obtained ZCIS QDs is higher than that of the core/shell structured CIS/ZnS QDs. This demonstrates a higher PL emission efficiency and lower trap state defects density for the obtained ZCIS QDs in comparison with that of CIS/ZnS core/shell structured QDs.

Accordingly, the cell performance also has a heavy dependence on the Zn/Cu–In ratio of the obtained ZCIS QD sensitizers (photovoltaic parameters of Zn–Cu–In–S QDSCs with various molar ratio of Zn/Cu–In are available in Table S1–S4 respectively in the ESI).† Experimental results indicate that ZCIS solar cell samples with Zn/Cu–In ratio of 0.4 achieved the best PCE. Hereafter, for the ZCIS QDSC, we will focus on the ZCIS QDs with Zn/Cu–In ratio of 0.4, and compared their performance with those of ZnS/CIS and CIS derived devices.

**Fig. 2:**
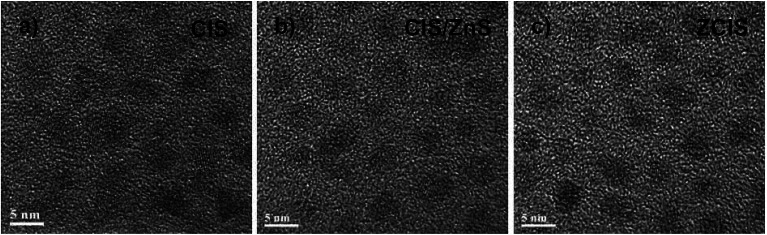
TEM images of (a) CIS; (b) CIS-Z, and (c) ZCIS QDs.

Transmission electron microscopy (TEM) images of three representative (CIS, CIS/ZnS and ZCIS) QD samples shown in Fig. 2 indicate that these QDs possess homogeneous size distribution with an average particle size about 4.0 nm. This result also demonstrates that the formation of core/shell structured or alloyed configuration does not vary the particle size even though the photoelectric properties been tuned. Furthermore, both the homogeneous size distribution and the small particle size of the obtained QD sensitizers favor the efficient immobilization of QDs on TiO_2_ mesoporous film electrode.

**Fig. 3:**
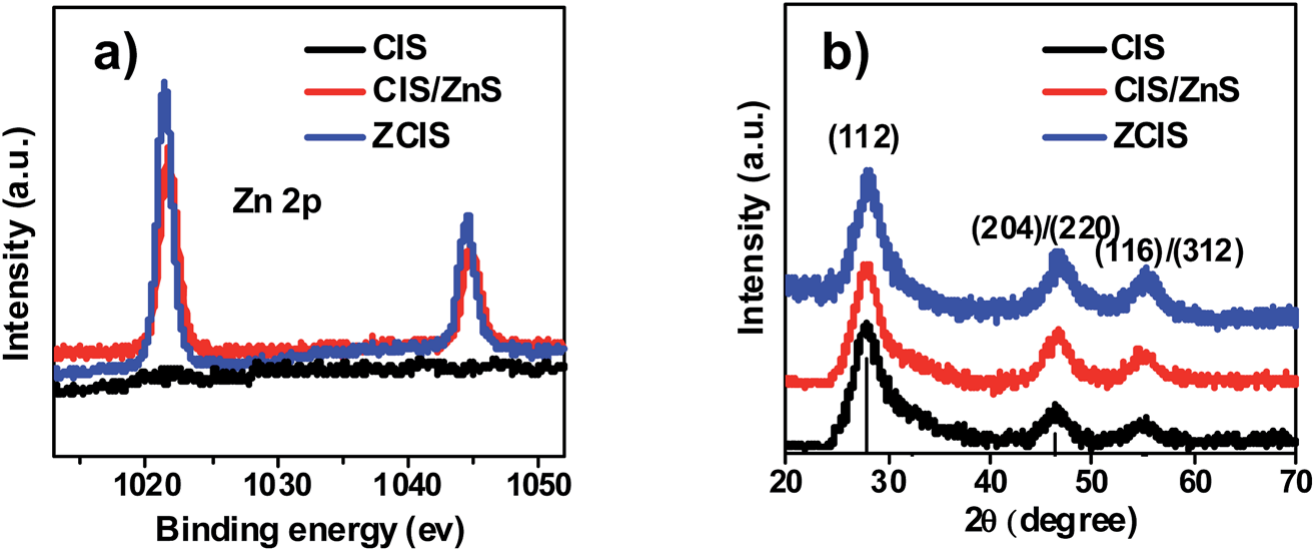
(a) XPS (Zn 2p peak) image of CIS, CIS/ZnS and ZCIS sensitizers electrode; (b) XRD patterns of CIS, CIS/ZnS and ZCIS QDs.

The successful incorporation of Zn precursor into CIS QDs was confirmed by the X-ray photoelectron spectroscopy (XPS) characterization. As shown in the XPS spectra for three QD samples (Fig. 3a), the Zn 2p peaks located at 1021 eV and 1044 eV were found be existed in CIS/ZnS and ZCIS QDs, respectively, while there is no Zn signal in the CIS QDs sample. This result supports convincing evidence for the incorporation of Zn component in the CIS/ZnS and ZCIS QD samples. The X-ray diffraction (XRD) pattern shown in Fig. 3b demonstrates a uniform chalcopyrite structure for the three QD samples without changing the crystalline form of sensitizers. In detail, the diffraction pattern was shifted to small angel along with the incorporation of Zn element in the CIS/ZnS and ZCIS QDs. This should be ascribed to the smaller crystalline parameters compared to that of CIS.^46,49^

### Photovoltaic performance

**Fig. 4:**
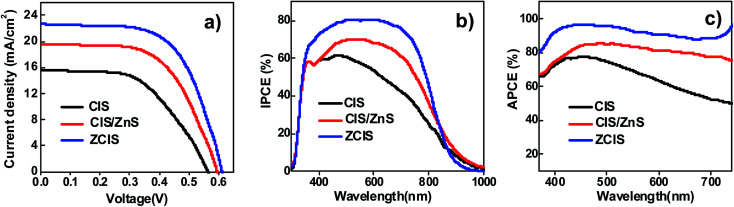
(a) *J*–*V* curves of the best solar cell based on CIS, CIS/ZnS and ZCIS sensitizers; (b) IPCE and (c) APCE characterization of CIS, CIS/ZnS and ZCIS QDSCs.

In order to evaluate the influence of QD sensitizers with different structural configurations on the photovoltaic performance of the resulting QDSCs, alloyed ZCIS, core/shell structured CIS/ZnS, and pristine CIS QD based QDSCs under their optimal conditions were constructed and their corresponding photovoltaic performances were measured. The obtained high-quality ZCIS, CIS/ZnS, and CIS QDs were immobilized on TiO_2_ mesoporous film electrode with thickness of 25.0 μm (Fig. S5†) with high surface coverage through the well-developed capping ligand induced self-assembly approach.^40^ In this sensitization approach, the as-prepared oil-soluble QDs were firstly transferred into water-soluble *via* a ligand exchange process with use of bi-functional mercaptopropionic acid (MPA) ligand. The obtained water-soluble MPA-capped QD sensitizers were then self-assembled onto TiO_2_ electrodes by pipetting QDs aqueous solution onto the oxide matrix and staying for 2 h. Absorption spectra of ZCIS, CIS/ZnS, and CIS sensitized TiO_2_ film electrodes are shown in Fig. S6† with the corresponding photographs of the films are shown in the inset. After deposition of ZnS passivation layer on the QD sensitized photoanodes, sandwich-type cells were constructed using Cu_2_S/brass as counter electrode and polysulfide electrolyte (2.0 M Na_2_S, and 2.0 M S in aqueous solution) as hole transporting media. The photoelectric performance of each type of QDSCs with five parallel samples was measured. The performance parameters and the current–voltage (*J*–*V*) curves for each type of QDSCs were shown in Table S5† and Fig. 4a, respectively. It is observed that the short-circuit current density (*J*_sc_) and fill factor (FF) of QDSCs based on ZCIS alloyed QDs are remarkably enhanced compared to those of CIS cell and have a significant improvement than those of CIS/ZnS derived QDSCs. Table 1 shows the average parameters for all kinds of solar cells. Notably, the QDSCs constructed by ZCIS alloyed QDs achieved an average PCE of 8.47% (*V*_oc_ = 0.613 V, *J*_sc_ = 22.62 mA cm^−2^, FF = 0.610), and the best PCE of 8.55%.

**Table 1:** Photovoltaic parameters of QDSCs based on different QD sensitizers

Cells	*V*_oc_ (V)	*J*_sc_ (mA cm^−2^)	FF	PCE (%)
ZCIS^a^	0.613	22.62	0.610	8.47 ± 0.05
CIS/ZnS^a^	0.601	19.73	0.586	6.95 ± 0.18
CIS^a^	0.565	15.48	0.533	4.66 ± 0.08
ZCIS^b^	0.612	22.70	0.615	8.55
CIS/ZnS^b^	0.602	19.83	0.596	7.12
CIS^b^	0.563	15.62	0.540	4.74

a Average value from five cells.

b Champion performance of different devices.

In order to explore the intrinsic reason for the enhancement of *J*_sc_ performance, incident photon-to-current conversion effective (IPCE) spectra and absorbed photon to electron conversion efficiency (APCE) spectroscopy characterizations were carried out for each kind of QDSCs, and corresponding results were shown in Fig. 4b and c. The IPCE value of ZCIS QDSCs increased significantly accompanied by a blue-shift of absorption in comparison to other two kinds of QDSCs. This is well matched with the variation of APCE value. Therefore, it is concluded that the increase of APCE and IPCE value was attributed to a higher charge collection efficiency (*η*_cc_) and/or electron injection efficiency (*ϕ*_inj_) excited in ZCIS QDs according to the corresponding theory reference (IPCE = LHE × *ϕ*_inj_ × *η*_cc_, LHE represents the light harvest efficiency). It can be reasonably concluded that an ascendant conduction band edge based on ZCIS sensitizer deriving from the construction of alloy QD structure elevates the energetic driving force for injecting photo-generated electrons into TiO_2_ receptor, therefore, resulting in a superior performance of photocurrent.

### Open-circuit voltage decay and impedance spectroscopy characterization

**Fig. 5:**
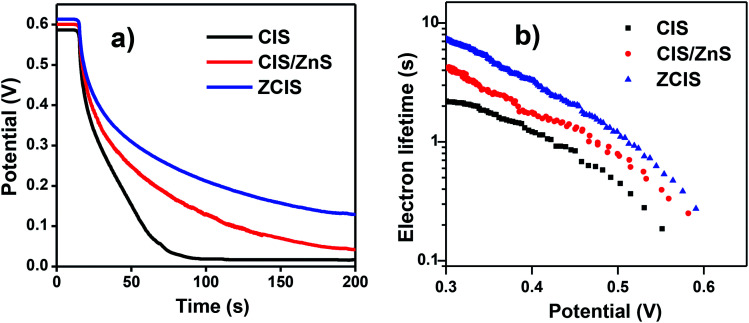
(a) Open-circuit voltage decay curves of cells based on different QDs sensitizers; (b) the calculated electron lifetimes.

Open-circuit voltage decay (OCVD) measurement was implemented to reflect the relevant information for electron recombination dynamics.^16,50,51^ As shown in Fig. 5a, the decay rate of *V*_oc_ based on ZCIS QDSCs is lower than that of the pristine CIS and CIS/ZnS derived cells. This indicates a lower charge recombination process existing in ZCIS cells. In addition, electron lifetime *τ*_n_ can be calculated according to the equation of *τ*_n_ = −(*k*_B_*T*/*e*)(d*V*_oc_/d*t*)^−1^, where *k*_B_, *T* and *e* represent the Boltzmann constant, the absolute temperature (298 K), and electronic charge, respectively. The corresponding *τ*_n_ curves are shown in Fig. 5b. It can be found that ZCIS QDs exhibits the longest electron lifetime (*τ*_n_) among three types of cell devices. The lower decay rate of *V*_oc_ and the longer electron lifetime signify that ZCIS alloy structure diminishes trap state density. This is effective to inhibit charge recombination at TiO_2_/QD/electrolyte interfaces, and therefore contributes to the improvement of photovoltage performance.

**Fig. 6:**
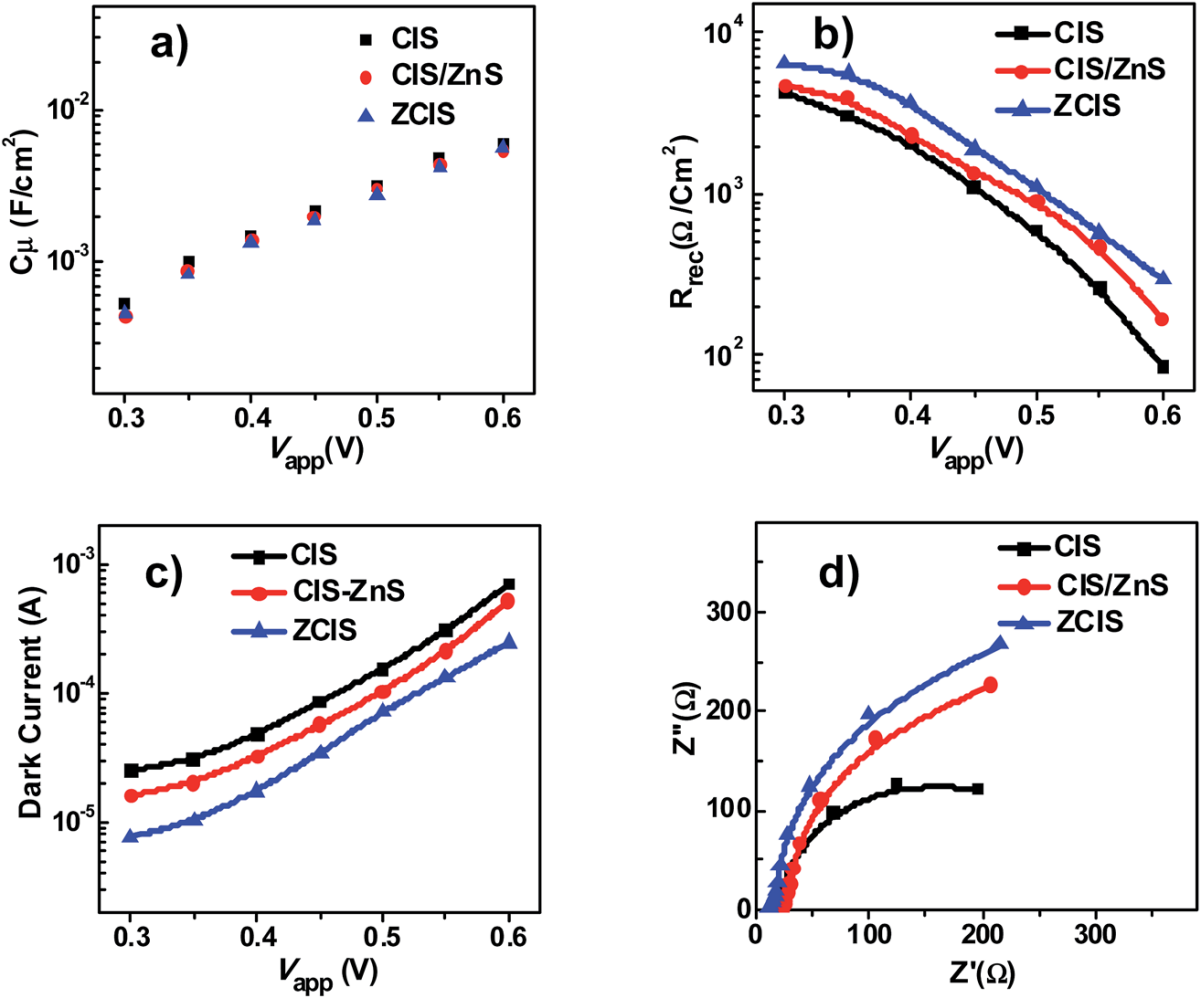
Analysis of parameters extracted from electrochemical impedance spectroscopy measurement of QDSCs based on various QDs (CIS, CIS/ZnS, and ZCIS). (a) Chemical capacitance *C*_μ_; (b) recombination resistance *R*_rec_; and (c) dark current at a series of forward bias; (d) Nyquist curves at the forward bias of −0.55 V.

Electrochemical impedance spectroscopy (EIS) measurement was performed to reveal the influence of the incorporation of Zn precursor into the synthesis of CIS QDs *via* alloy procedure on charge recombination process.^16,50,52,53^ EIS characterization was measured under dark condition with forward bias ranging from −0.30 to −0.60 V. The EIS curves based on different cells at an applied voltage bias were presented in Fig. S8.† The chemical capacitance (*C*_μ_) and recombination resistance (*R*_rec_) were extracted by fitting the obtained EIS data under various forward bias with an equivalent-circuit model. In Fig. 6a, the three types of QDSCs with use of different QD sensitizers showed nearly identical *C*_μ_ value. The observed same *C*_μ_ value implied that the diversity of sensitizer serving as photoelectron harvesting material has no influence on the position of conduction band or inherent electron density in TiO_2_ substrate. However, from Fig. 6b it is observed that *R*_rec_ values of solar cell based on ZCIS QDs surpasses significantly that of the pristine CIS and CIS/ZnS cells, and the enhancement of recombination resistance would be accredited as the restraint of charge recombination at the interfaces of QD/TiO_2_ or QD/electrolyte. For clarity, Fig. 6c gives a comparison of dark current among three diverse cells under an applied forward bias ranging from −0.3 V to −0.6 V. Experiment results indicated that ZCIS cell possesses a superior performance of dark current, thus it further testifies that the charge recombination process from photoanode to electrolyte was inhibited efficiently. In addition, Nyquist plots (Fig. 6d) at certain forward bias (−0.55 V) for different cell devices are consistent with the variation trend of *R*_rec_ value as shown in Fig. 6b. This demonstrates that the incorporation of Zn element contributes to inhibition on the rate of unwanted electron loss at TiO_2_/QD/electrolyte interfaces. Meanwhile, the calculated electron lifetime (*τ*_n_ = *C*_μ_ × *R*_rec_) for ZCIS QDSC in Table 2 is superior to those based on CIS and CIS-Z cell, and thus also indicates the better blockage of charge recombination. The EIS characterization result is also well matched with *J*–*V* curve performance and result of OVCD measurement analysis (Fig. 5).

**Table 2:** EIS parameters extracted from the Nyquist plots of QDSCs based on CIS, CIS/ZnS, and ZCIS QDs under a forward bias voltage of −0.55 V

Cell	*R*_s_ (Ω cm^2^)	*R*_CE_ (Ω cm^2^)	*C*_μ_ (mF cm^2^)	*R*_rec_ (Ω cm^2^)	*τ*_n_ (ms)
CIS	17.33	5.94	3.95	258.6	1021.5
CIS/ZnS	23.00	5.62	4.33	464.8	2012.6
ZCIS	10.85	6.48	4.19	568.0	2379.9

**Fig. 7:**
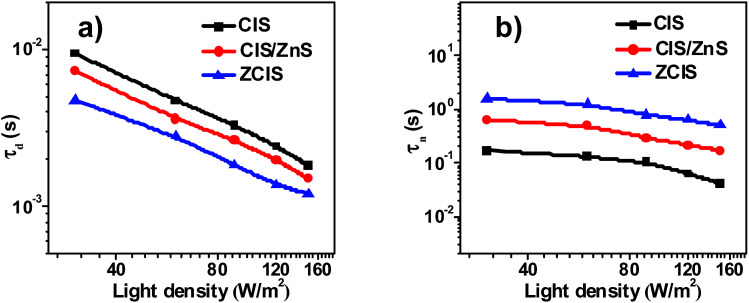
IMPS and IMVS characterization of solar cell based on CIS, CIS/ZnS and ZCIS QDs: (a) electron transient time (*τ*_d_); (b) electron time (*τ*_n_).

The measurement of Intensity-modulated photocurrent and photovoltage spectra (IMPS/IMVS) was carried out under a modulated homogeneous light to investigate charge recombination and electron transport mechanism. During the characterization of IMPS and IMVS, the calculated electron transient time (*τ*_d_) and electron time (*τ*_n_) reflect on the photovoltage and photocurrent according to the relative equation (*τ*_d_ = 1/2π*f*_IMPS_, *τ*_n_ = 1/2π*f*_IMVS_), where *f*_IMPS_ and *f*_IMVS_ represent the corresponding frequency at minimum position for IMPS and IMVS spectrum.^54–56^ As observed in Fig. 7a, the *τ*_d_ values of ZCIS solar cell (about 1.2–4.8 ms) displays the shortest degressive tendency among all three types of cells, where *τ*_d_ values for CIS and CIS-Z cell vary from 1.8 ms to 9.5 ms and 1.5 ms to 7.3 ms respectively. The better *τ*_d_ performance of ZCIS QDSC indicates a superior and efficient procedure for electron transport in contrast to that of CIS and CIS-Z cells. The measurement of IMVS (Fig. 7b) was obtained at variable light density (30–150 W m^−2^), as a result, a good agreement was reached between variation of *τ*_n_ and the EIS electron lifetime (Table 2), which further demonstrates that alloying Zn into CIS QD renders depression of charge recombination and thus achieves a better performance of photovoltage.

## Conclusions

In summary, quaternary ZCIS alloyed QDs was successfully synthesized by a simultaneous nucleation and growth strategy, and used as light harvesting sensitizers in the construction of QDSCs. The obtained ZCIS QD based QDSCs exhibited superior photovoltaic performance in comparison with the pristine CIS QDs, and core/shell structured CIS/ZnS QDs based cell devices. In addition, the Zn content in ZCIS QDs has a significant influence on the photovoltaic performance of the resulting cells. The average PCE of the cells based on ZCIS sensitizer was enhanced by 21%, and 82% in comparison to those of CIS/ZnS, and CIS solar cells, respectively. The formation of ZCIS alloy structure favors the suppression of charge recombination lost as well as acceleration of electron injection from QD to TiO_2_ electron acceptor. Meanwhile, the efficient suppression of unwanted charge recombination in ZCIS QDSCs was confirmed by APCE, OCVD, EIS, IMPS & IMVS characterizations.

## Conflicts of interest

There are no conflicts of interest to declare.

## Supplementary Material

RA-008-C7RA12321C-s001
