# Supplementary material for: Comparative advantages of Zn–Cu–In–S alloy QDs in the construction of quantum dot-sensitized solar cells
Source: RSC Adv. 2018 Jan 18;8(7):3637–45. doi: 10.1039/c7ra12321c (PMC9077672; doi:10.1039/c7ra12321c)
Supplement: RA-008-C7RA12321C-s001 [file RA-008-C7RA12321C-s001.pdf]

Electronic Supplementary Material (ESI) for RSC Advances.
This journal is © The Royal Society of Chemistry 2018

Electronic Supporting Information (ESI)

Comparative advantages of Zn-Cu-In-S alloy QDs in the construction of quantum dot-sensitized solar cells

Liang Yue,[ab] Huashang Rao,[b] Jun Du,[a] Zhenxiao Pan,*[b] Juan Yu[a] and Xinhua Zhong*[a]

[a]School of Chemistry and Molecular Engineering, East China University of Science and Technology, Shanghai 200237, China

[b]College of Materials and Energy, South China Agricultural University, 483 Wushan Road, Guangzhou 510642, China

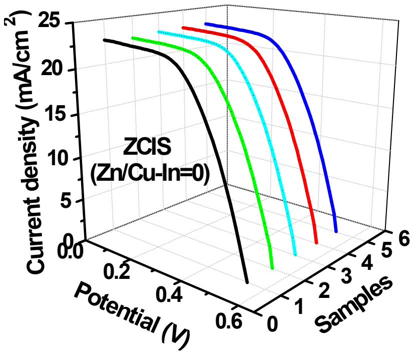

Fig. S1 *J-V* curves of five cells in parallel for ZCIS QDSCs synthesized with a molar ratio (Zn/Cu-In = 0).

Table S1 Photovoltaic parameters of CIS QDSCs under the illumination of 1 full sun intensity (AM 1.5 G, 100 mW cm^{-2}).

V_{oc} (V)	J_{oc} (mA/cm^2)	FF	PCE (%)
0.528	22.62	0.545	6.51
0.535	22.56	0.550	6.64
0.530	22.58	0.553	6.62
0.536	22.37	0.555	6.65
0.526	22.56	0.545	6.47

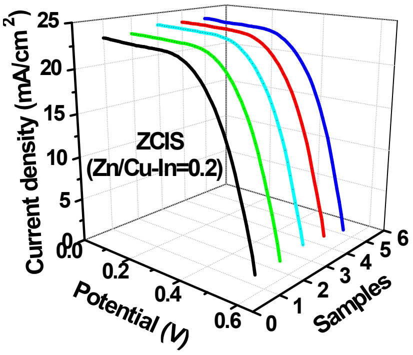

Fig. S2 *J-V* curves of five cells in parallel for ZCIS QDSCs synthesized with a molar ratio (Zn/Cu-In = 0.2).

Table S2 Photovoltaic parameters of ZCIS QDSCs synthetized with a molar ratio (Zn/Cu-In = 0.2) under the illumination of 1 full sun intensity (AM 1.5 G, 100 mW cm^{-2}).

Zn amount	V_{oc} (V)	J_{oc} (mA/cm^2)	FF	PCE (%)
	0.572	23.55	0.582	7.84
	0.575	23.63	0.585	7.95
0.2	0.576	22.87	0.583	7.68
	0.575	23.56	0.578	7.83
	0.578	23.61	0.579	7.90

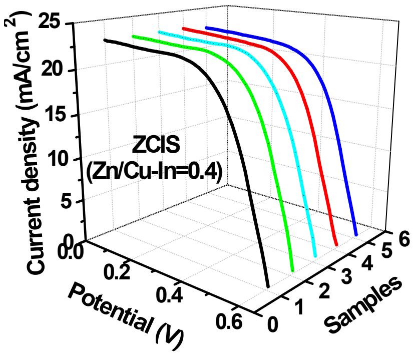

Fig. S3 *J-V* curves of five cells in parallel for ZCIS QDSCs synthesized with a molar ratio (Zn/Cu-In = 0.4).

Table S3 Photovoltaic parameters of ZCIS QDSCs synthetized with a molar ratio (Zn/Cu-In = 0.4) under the illumination of 1 full sun intensity (AM 1.5 G, 100 mW cm^{-2}).

Zn amount	V_{oc} (V)	J_{oc} (mA/cm^2)	FF	PCE (%)
	0.611	22.74	0.606	8.42
	0.610	22.72	0.608	8.43
0.4	0.602	22.68	0.620	8.48
	0.612	22.75	0.605	8.43
	0.600	22.59	0.620	8.39

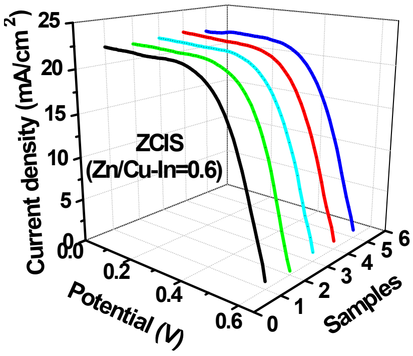

Fig. S4 *J-V* curves of five cells in parallel for ZCIS QDSCs synthesized with a molar ratio (Zn/Cu-In = 0.6).

Table S4 Photovoltaic parameters of ZCIS QDSCs synthetized with a molar ratio (Zn/Cu-In = 0.6) under the illumination of 1 full sun intensity (AM 1.5 G, 100 mW cm^{-2}).

Zn amount	V_{oc} (V)	J_{oc} (mA/cm^2)	FF	PCE (%)
	0.605	21.86	0.611	8.08
	0.604	21.93	0.595	7.88
0.6	0.605	22.00	0.604	8.04
	0.606	21.98	0.602	8.02
	0.606	21.48	0.613	7.98

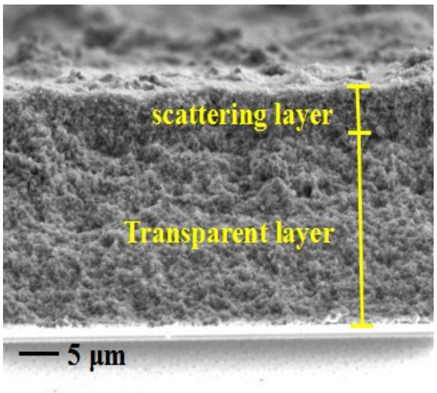

Fig. S5 Cross section SEM image of the TiO_2 photoanode film.

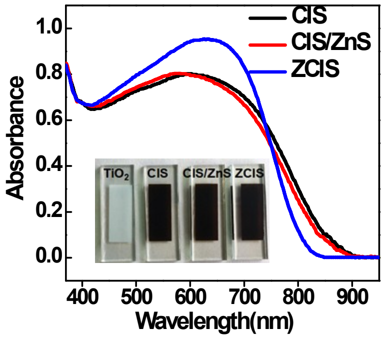

Fig. S6 UV-vis absorption spectra of CIS, CIS/ZnS and ZCIS QDs deposited on TiO_2 mesoporous film.

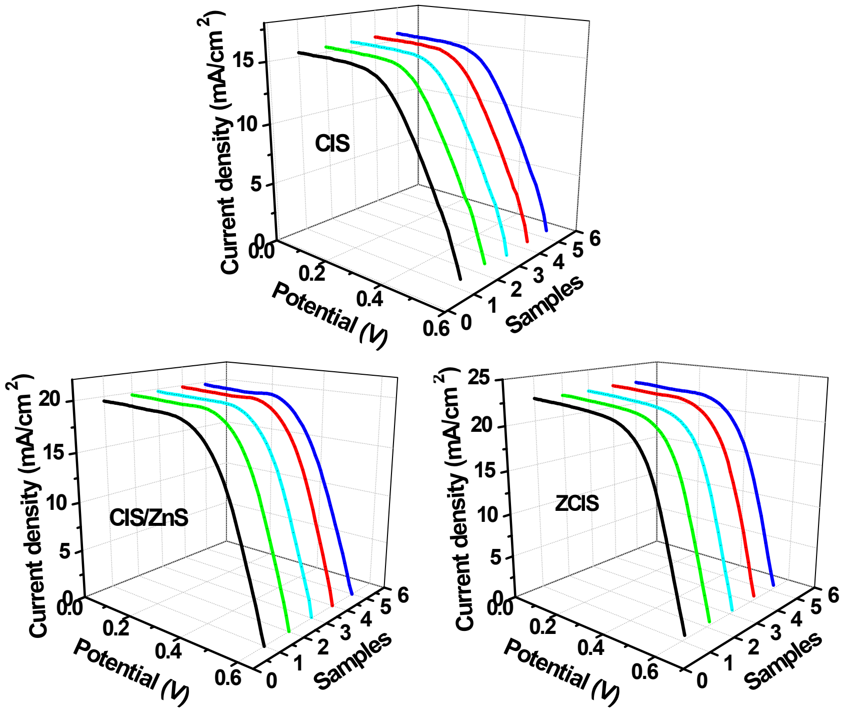

Fig. S7 *J-V* curves of five cells in parallel for individual CIS, CIS/ZnS and ZCIS QDSCs based on Cu_2S counter electrodes.

Table S5 Individual and average photovoltaic parameters of CIS, CIS/ZnS and ZCIS based QDSCs under the illumination of 1 full sun intensity (AM 1.5 G, 100 mW cm^{-2}).

Samples	V_{oc} (V)	J_{sc} (mA/cm^2)	FF	PCE (%)
	0.566	15.30	0.526	4.56
	0.563	15.55	0.541	4.73
CIS	0.569	15.52	0.527	4.65
	0.565	15.41	0.531	4.62
	0.563	15.62	0.540	4.74
Average	0.565 ± 0.003	15.48 ± 0.13	0.533 ± 0.007	4.66 ± 0.08
	0.599	19.58	0.586	6.87
	0.603	19.72	0.589	7.00
CIS/ZnS	0.600	19.78	0.597	7.09
	0.602	19.83	0.596	7.12
	0.601	19.73	0.564	6.68
Average	0.601 ± 0.02	19.73 ± 0.09	0.586 ± 0.01	6.95 ± 0.18
	0.612	22.75	0.605	8.43
	0.614	22.41	0.615	8.47
ZCIS	0.611	22.75	0.606	8.42
	0.612	22.70	0.615	8.55
	0.616	22.50	0.611	8.48
Average	0.613 ± 0.002	22.62 ± 0.14	0.610 ± 0.005	8.47 ± 0.05

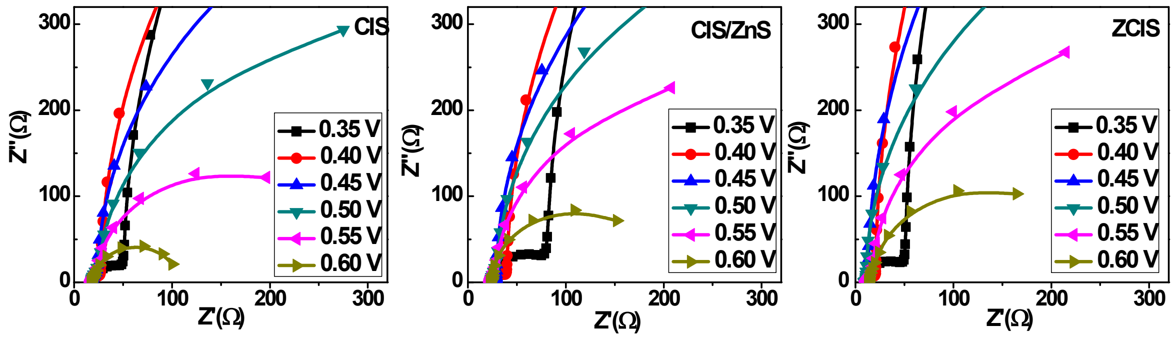

Fig. S8 Nyquist curves for CIS (a); CIS/ZnS (b) and ZCIS (c) based QDSCs at different forward bias.
